# MiR-124 inhibits the migration and invasion of human hepatocellular carcinoma cells by suppressing integrin αV expression

**DOI:** 10.1038/srep40733

**Published:** 2017-01-17

**Authors:** Qian Qian Cai, Yi Wei Dong, Rong Wang, Bing Qi, Jun Xia Guo, Jing Pan, Yuan Yuan Liu, Chun Yi Zhang, Xing Zhong Wu

**Affiliations:** 1 Department of Biochemistry and Molecular Biology, School of Basic Medical Sciences, Fudan University, Key Lab of Glycoconjugate Research, Ministry of Public Health, Shanghai, PR. China; 2 Yu Ying Children’s Hospital, Wenzhou Medical University, Wenzhou, PR. China

## Abstract

Tumor metastasis is the major cause of cancer-related death especially in human hepatocellular carcinoma (HCC). Although microRNAs have been implicated in tumor development, the roles of miR-124 in HCC metastasis are still not well understood. We conducted functional analysis in this study to investigate miR-124. We observed that miR-124 significantly retarded the wound healing and migration of HCC SMMC-7721 and BEL-7404 cells. Further analysis indicated miR-124 directly targeting the transcriptional factor Sp1 which is an important transcription factor for the integrin αV subunit gene transcription. Co-transfection of miR-124 with the luciferase reporter containing Sp1 3′ untranslated region (UTR) significantly suppressed the luciferase activities. While mutation of the binding site of miR-124 in Sp1 mRNA 3′UTR completely abrogated the suppression of miR-124. Overexpression of miR-124 resulted in robust downregulation of Sp1 and integrin αV expression at either mRNA or protein level. Ectopic expression of miR-124 in HCC dramatically repressed the wound healing and migration *in vitro* and tumor metastasis in mouse experiments. Our findings demonstrated that miR-124 played as an important role in regulation of integrin αV expression in HCC, and reintroduction of miR-124 might be an alternative therapeutic strategy for controlling integrin αV expression in HCC.

MicroRNA (miR) is a single-stranded, non-coding RNA molecule of 22–25 nucleotides, which are a family of regulatory molecules involved in controlling gene expression, translation and cellular biological behaviors, even in tumorigenesis. MiR-124 was first identified by cloning studies in mice^1^ and is most abundant, well-conserved and specific microRNA in the brain^2^. In recent years, some studies indicated that the CPG island (or CG site) methylation of miR-124 gene is associated with the advanced tumors and the recurrence in patients with renal carcinoma^3^. MiR-124 is considered as one of the expression-silenced miRNAs in tumors including gastric cancer cells^4^, breast cancer cells^5^ and nasopharyngeal carcinoma^6^. MiR-124 was also one of the down-expressed microRNAs in human hepatocellular carcinoma (HCC)^7^.

Hepatocellular carcinoma (HCC) is one of the most malignant tumors with poor prognosis largely due to remote metastasis and postsurgical recurrence. There are more than half a million newly diagnosed patients each year. The tumor progression and metastasis are the major cause of cancer-related deaths in patients with HCC. During the process of tumor invasion and metastasis, integrins act as crucial transducers of bidirectional cell signaling, regulating cell adhesion, migration, and tissue remodeling^8^,^9^. Integrins are a family of transmembrane adhesion receptors composed of 18α and 8β subunits that interact non-covalently to form 24 different heterodimeric receptors^10^. Because integrins are the primary receptors to extracellular matrix (ECM) molecules for cellular adhesion, the heterodimer integrin on the cell surface allows cells to recognize and responds to a variety of signals from ECM environments. Activation and elevated expression of integrin-coupled signaling effectors have been implicated in the induction of angiogenesis and metastasis of human cancers^11^,^12^. Overexpressed integrin αVβ3 forms the oncogenic unit with Src to promote tumor cell proliferation and drives the malignance and stemness of tumor cells^13^,^14^. Integrin αVβ3 antagonists have shown encouraging anti-tumor effect on glioma in the initiatory trials^15^. Overexpressed integrin αVβ3 helps glioblastoma cells to escape senescence by activation of the cytoskeletal regulatory kinase PAK4^16^ and promotes the migration of HCC^17^. However the dysregulation of integrin αVβ3 in cancer cells especially in HCC is not well understood. The subunit of integrin αV is encoded in the gene of ITGAV. Specificity protein 1 (Sp1) belongs to the specificity protein/Krüppel-like factor family of transcription factors that recognizes and binds, via three Cys2-His2 zinc finger motifs localized at its carboxyl terminus, GC-rich sites, thus regulating the transcription of target genes^18^. The promoter region of ITGAV gene encoding integrin αV subunit, contains four GC-rich motifs. In our previous study^19^ we observed that Sp1 bound to integrin αV subunit gene promoter and activated its transcription in the pathway of integrin αV transcription regulated by sulfatide. In this study, we tried to explore further the mechanism of sulfatide inducing integrin αV subunit gene expression on the base of our previous results. In analysis of integrin αV regulation of cell migration in HCC we identified miR-124 as a novel regulator of the pathway, targeting Sp1 directly in HCC. Moreover, overexpression of miR-124 inhibited invasion and metastasis of HCC through integrin αV subunit implicated.

## Results

### MiR-124 suppresses the wound healing and migration capability of HCC

**Figure 1:**
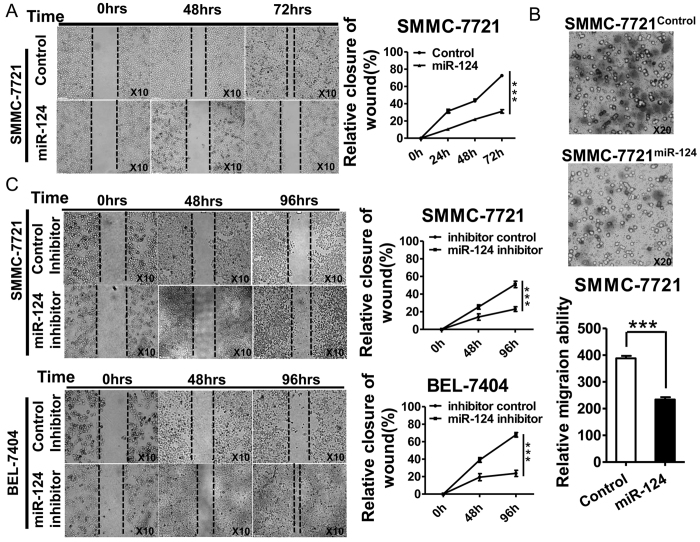
MiR-124 suppresses the wound healing and migration of HCC. (**A**) Representative micrographs of wound closure and quantitative analysis in HCC cells with miR-124 overexpression and control. Original magnification:10×. (**B**) Representative micrographs of transmembrane migration and quantitative analysis. Original magnification:20×. (**C**) Representative micrographs of wound closure and quantitative analysis in miR-124 inhibitor and control groups. Original magnification:10×. Data are representative of three independent experiments. ***p < 0.001.

In order to investigate the roles of miR-124 in cell migration and metastasis-related behaviors, we performed the wound healing assay and transmembrane migration assay. The miR-124 expressional construct was transfected into human hepatocellular carcinoma cells of either SMMC-7721 or BEL-7404 cells, to achieve over-expression of miR-124. 72 hours after wound healing in SMMC-7721 cells, the scratched wound in miR-124 group was not closed as fast as the control group. The relative closure in miR-124 group was significantly slower than control (Fig. 1A). Moreover, the migrated cells through the polyporous membrane were significantly fewer in miR-124 group than control cells (Fig. 1B). The invasion capability of SMMC-7721 cells transfected with miR-124 through Matrigel in miR-124 group was lower than control (data not shown). To confirm the role of miR-124 in migration further, both SMMC-7721 and BEL-7404 cells were transfected with specific miR-124 inhibitor. As a result, the group of antagomirs showed significantly faster wound healing compared to control group in both cell lines (Fig. 1C). These suggested that miR-124 suppressed cell migration in HCC.

**Figure 2:**
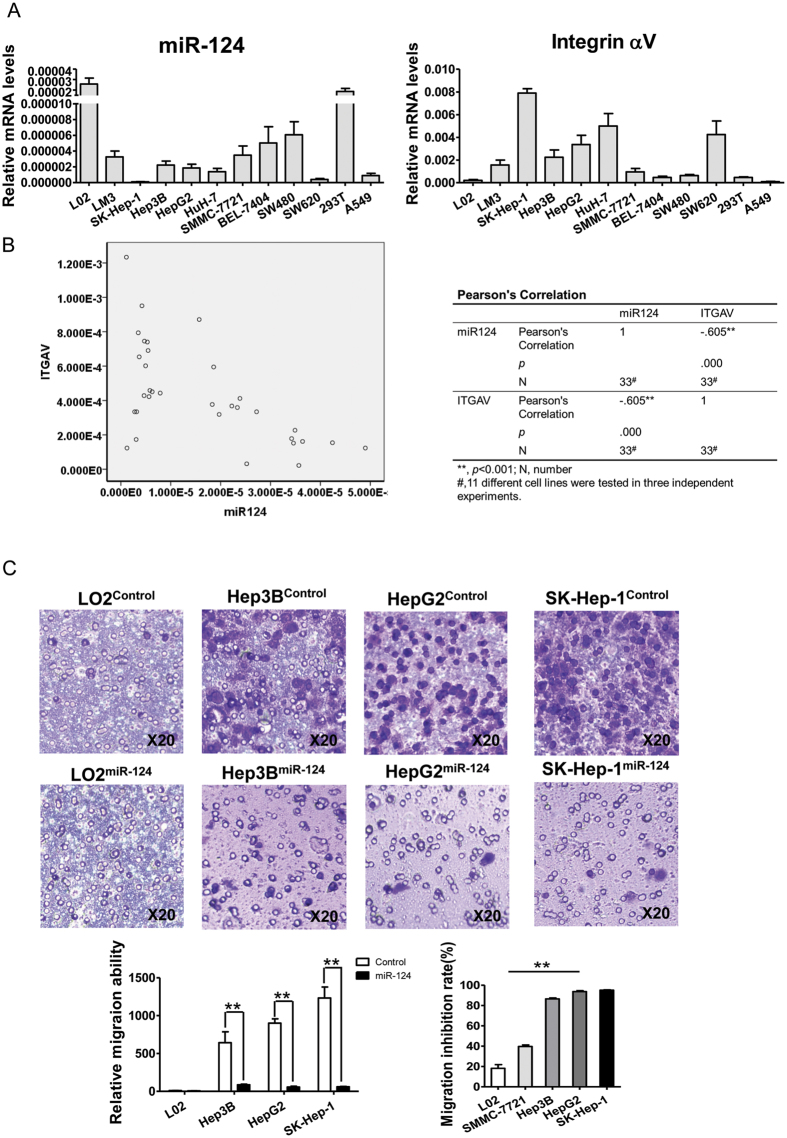
Correlation analysis between miR-124 and ITGAV expressions. (**A**) The expression levels of miR-124 and ITGAV were measured by qPCR in various cells. (**B**) Pearson correlation analysis of miR-124 and ITGAV expression in A. The spot represents the value of miR-124 and ITGAV expression (left). Correlation analysis is summarized on the right. (**C**) Representative micrographs of transmembrane migration (top) and quantitative analysis (bottom left). Original magnification: 20×. Comparison analysis of inhibitory rate was made between the cells with high and low expression levels of integrin αV(bottom right).

Since cell migration is usually controlled by integrins, we then analyzed the relationship between miR-124 and integrin αV expression. The miR-124 and integrin αV expression levels were measured in 11 different non-tumor and tumor cell lines with qPCR (Fig. 2A). We interestingly observed that the expression level of integrin αV subunit was often low when miR-124 level was high. A strong correlation was noted between miR-124 and integrin αV subunit expression levels with the correlation coefficient −0.605 (*P* < 0.01) (Fig. 2B), indicating that miR-124 was associated with the down regulation of integrin αV subunit expression. Further in Hep3B, HepG2 and SK-Hep-1 cells which expressed high levels of integrin αV, the inhibitory effect on the cell migration by miR-124 was investigated (Fig. 2C). The inhibitory rate in Hep3B, HepG2 and SK-Hep-1 cells was 86%, 93% and 95%, more robust than those (40%) in SMMC-771 cells (Fig. 2C bottom right). However the inhibition in LO2 cells was slight since the basal levels of migration and integrin αV expression were quite low.

### MiR-124 directly regulates the transcription factor Sp1

**Figure 3:**
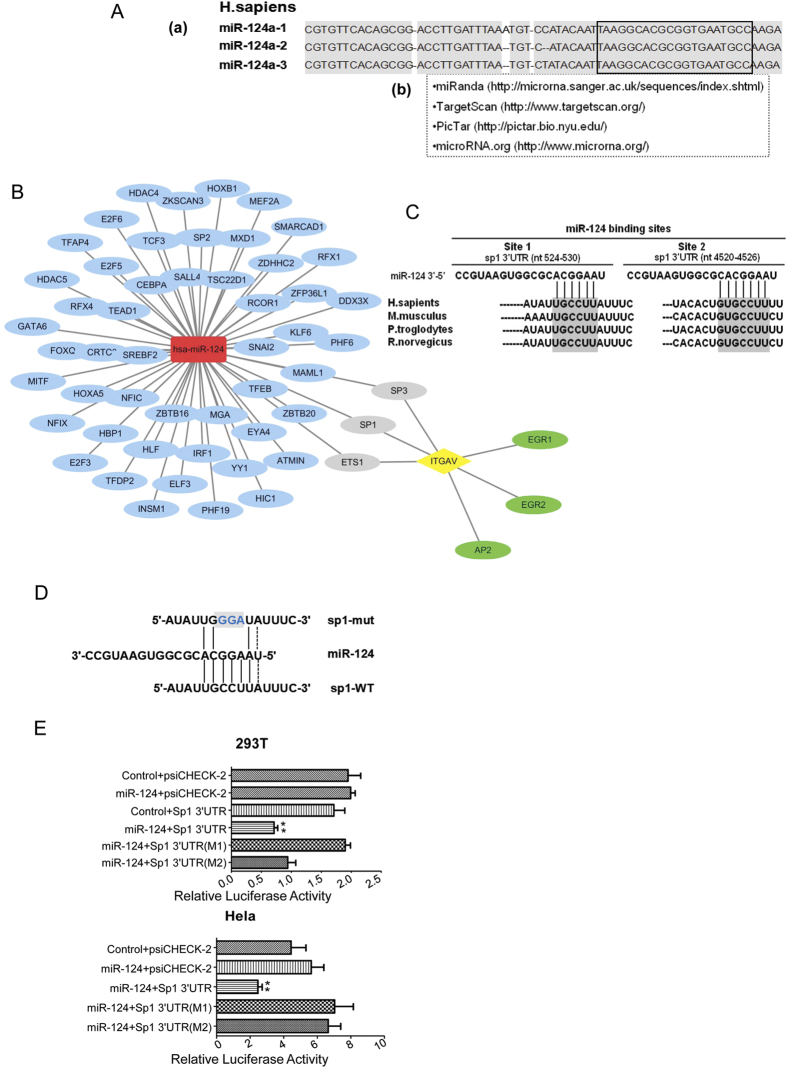
MiR-124 regulates the transcription factor Sp1. (**A**) The sequences of human miR-124-1, 2, 3 genomic loci (a). Framed part indicates the mature sequence. Four online algorithms were used to predicate miR-124 targets (b). (**B**) Network of predicated miR-124-targeted transcription factors and the association with ITGAV. The red rectangle indicates miR-124, the yellow diamond indicates ITGAV gene, and the blue circles indicate the miR-124-targeted TF mRNAs. The TFs linking to ITGAV have specific binding sites on ITGAV promoter. (**C**) Sp1 has two conserved miR-124 binding sites within 3′UTR. (**D**) The schematic of miR-124 binding site 1 mutation (M1) of Sp1 3′UTR. (**E**) Reporter luciferase activities were measured in HEK-293T (upper) and HeLa (lower) cells. Data are representative of three independent experiments. **p < 0.01.

MiR-124 is encoded in three genomic loci [miR-124a-1 (8p23.1), miR-124a-2 (8q12.3), and miR-124a-3 (20q13.33)], but these three miR-124a loci give rise to only one mature miRNA, miR-124 (Fig. 3A top). Having found that miR-124 inhibited wound healing and migration which were related to down-regulating integrin αV subunit, we next investigated the possible regulation of integrin αV subunit expressions by miR-124. As miRNA functions in the post-transcriptional regulation of gene expression by targeting 3′ untranslated region (UTR) of mRNAs, we analyzed and screened the possible target by bioinformatics. The predicated mRNA target for mature miR-124 was analyzed using four online algorithms including miRanda, TargetScan, PicTar and microRNA.org (Fig. 3A bottom). The analysis results indicated that there were 53 transcription factors that either matched miR-124 target or were expressed in HCC. Among them Sp1, Sp3 and ETS1 were associated with integrin αV subunit gene transcription (Fig. 3B). Sp1 as an important transcription factor for integrin αV gene transcription was demonstrated in our previous study^19^. Sp1 3′UTR sequence possesses two conserved binding motifs for miR-124, which are well conserved from worm to human being (Fig. 3C). In order to confirm that miR-124 directly targets Sp1 3′UTR, we performed luciferase reporter assay. The wild-type full length of Sp1 mRNA 3′UTR was cloned into the downstream of the Renilla luciferase gene in the psiCHEC^TM^-2 Vector with a firefly luciferase coding gene as internal control. Besides, two mutant vectors contained 3 mutated bases in the predicted binding sites were constructed (Fig. 3D). HEK-293T and HeLa cells were co-transfected with these reporter constructs and miR-124 or control vectors. The group transfected with miR-124 rather than control vector significantly suppressed the luciferase activity of reporter genes containing 3′UTR of Sp1 in both cells. However, the suppression was completely abrogated in both HEK-293T and HeLa cells when the binding site 1 was mutated (Fig. 3E). The mutation of the binding site 2 resulted in the recovery of the reporter activity in HeLa cells. These results indicated that miR-124 directly targeted Sp1 mRNA via the putative binding sites in the 3′UTR.

### MiR-124 inhibits integrin αV expression

**Figure 4:**
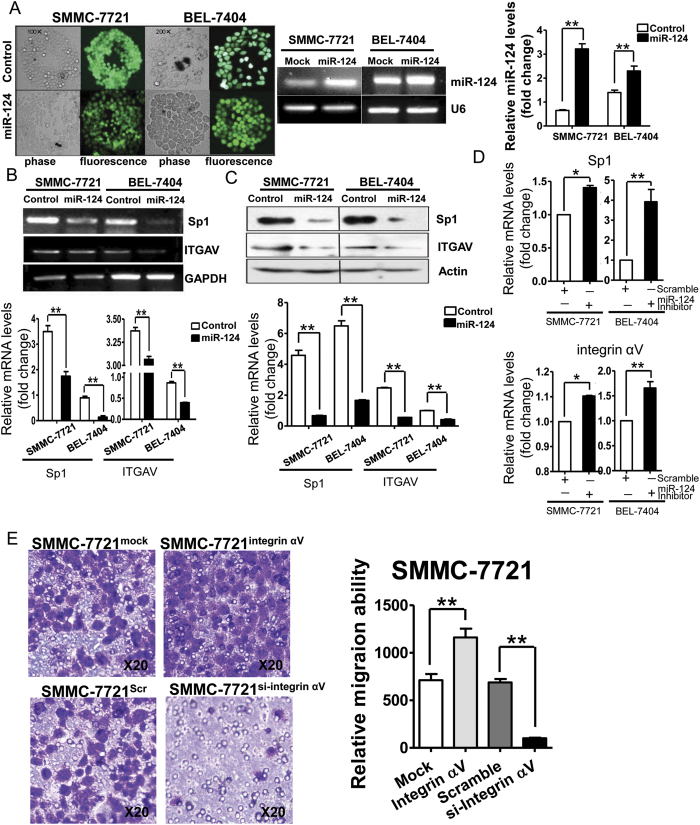
MiR-124 inhibits integrin αV subunit expression. (**A**) Representative micrographs of phase contrast and fluorescence microscope in SMMC-7721 and BEL-7404 cells infected by miR-124 or control lentivirus carrying GFP (left). Expression of miR-124 was measured by RT-PCR (middle) and quantitative RT-PCR (right). (**B**) Expression measurement of Sp1 and integrin αV by RT-PCR (upper) and quantitative analysis (lower). (**C**) Expression measurement of Sp1 and integrin αV by WB (upper) and quantitative analysis (lower). (**D**) Expression measurement of Sp1 and integrin αV by qRT-PCR in cells transfected with miR-124 inhibitor. (**E**) Representative micrographs of transmembrane migration and quantitative analysis. Original magnification:20×. Data are representative of three independent experiments. **p < 0.01.*p < 0.05.

Sp1 is an important transcription factor for integrin αV subunit gene^19^, and the down-regulation of Sp1 by miR-124 might lead to the down-regulation of integrin αV subunit gene transcription. In order to confirm miR-124 regulation of integrin αV expression, we investigated and observed the mRNA and protein expression levels of Sp1 and integrin αV subunit in SMMC-7721 and BEL-7404 cells with over-expression of miR-124. SMMC-7721 and BEL-7404 cells were infected with miR-124 or control lentivirus which is an efficient, stable gene delivery tool in mammalian cells to induce stable gain- and loss-of-function phenotypes for individual miRNAs^20^ or shRNAs^21^. The successful infection of miR-124 lentivirus was observed with fluorescence microscope (Fig. 4A) and the over-expression of miR-124 was validated in the miR-124 group by RT-PCR measurement (Fig. 4A middle & right). In these cells the expression of both Sp1 and integrin αV was then analyzed. The results of RT-PCR and Western-blotting showed that in the cells with over-expression of miR-124, the expression levels of Sp1 and integrin αV subunit reduced significantly in both SMMC-7721 and BEL-7404 cells (Fig. 4B and C). The mRNA expression levels of Sp1 and integrin αV reduced almost by 50% and 14% respectively in SMMC-7721 cells, and 84% and 53% in BEL-7404 cells. The protein levels of expression of Sp1 and integrin αV also reduced in miR-124 group by 85% and 74% in SMMC-7721, and 77% and 58% in BEL-7404 cells compared to control group. After transfection of anti-miR-124 inhibitor in SMMC-7721 and BEL-7404 cells, the expression of miR-124 was inhibited, and the expression levels of Sp1 and integrin αV subunit were simultaneously increased by 1.4 and 1.1 folds in SMMC-7721 cells, and 4 and 1.6 folds in BEL-7404 cells compared to negative control group (Fig. 4D). The overexpression of integrin αV significantly stimulated the cell migration ability, while integrin αV knockdown had a significant inhibitory effect (Fig. 4E). These suggested that ectopic expression of miR-124 contributed to the down-regulation of integrin αV subunit gene expression.

### MiR-124 inhibits HCC metastasis *in vivo*

**Figure 5:**
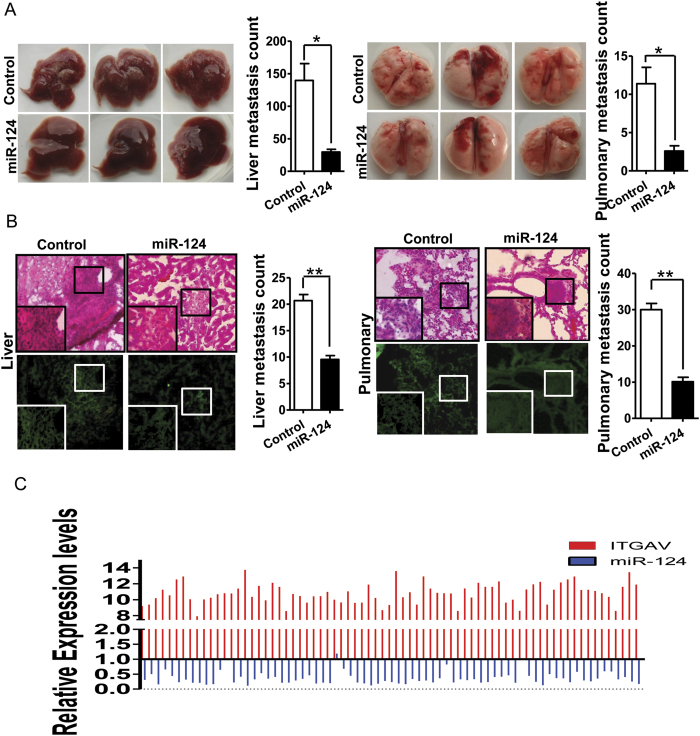
MiR-124 inhibits HCC metastasis *in vivo*. (**A**) Representative images of the livers (left) and lungs (right) from nude mice 4 weeks after injections and quantitative analysis of metastasis foci. *p < 0.05. (**B**) Representative images of H&E-stained sections and quantification of liver (left) and pulmonary (right) micro-metastases. **p < 0.01. (**C**) Comparison analysis of miR-124 and ITGAV expression in 58 human hepatocellular carcinoma cases.

Since miR-124 overexpression resulted in downregulation of integrin αV subunit expression, then animal experiments were performed to evaluate the influence of miR-124 overexpression on HCC metastasis *in vivo*. We employed the SMMC-7721 cells stably expressing miR-124 and green fluorescence protein (GFP) as the cell model for *in vivo* metastasis studies. Four weeks after the injection by tail vein of nude mice, the metastasis foci were examined at the liver and lung of both miR-124 and control groups. The hepatic metastasis and pulmonary metastases were first examined through organ anatomy and then pathologic examination. Strikingly, the group of mice receiving miR-124 transfection showed significantly fewer metastases colonies in the liver and lung by 79% and 77% respectively. However, the control group showed significant more hepatic and pulmonary metastases foci (Fig. 5A). Parallel with the anatomy results, H&E staining of lungs also showed that the number and size of micro-metastases foci were significantly more and larger in control group than those in miR-124 group (p < 0.001, Fig. 5B). These results indicated that miR-124 was capable of suppressing HCC metastasis *in vivo*.

We also analyzed human hepatocellular carcinoma gene expression data from TCGA (The Cancer Genome Atlas), in which 58 cases included both mRNA expression and miRNA expression data. Interestingly, all of these cases showed decreased miR-124 expression except 3 cases. While integrin αV subunit expression levels were elevated in all these cases (Fig. 5C). This implied negative association of miR-124 with integrin αV expression levels.

## Discussion

More and more studies have revealed that miRNAs abnormally expressed in human cancers involve in tumor progression as either oncogenes or tumor suppressors^22^,^23^. Moreover, the abnormal expression of miRNAs may have molecular functional links with the malignant hallmarks such as aberrant cell growth, angiogenesis, invasion and metastasis^24^. Hence, investigation of miRNA regulation network could help us understand the progression of tumor diseases and stratify their prognostic risks. MiR-124 is abundantly expressed in the brain tissues, but the roles of miR-124 were also noted in gastric cancer^4^ and nasopharyngeal carcinoma^6^. In this study, we demonstrated that miR-124 was involved in the regulation of migration and metastasis of human hepatocellular carcinoma cells (SMMC-7721 and BEL-7404). It was noted in our study that miR-124 played an important negative role in regulation of migration and metastasis of HCC cells. MiR-124 significantly slowed the wound healing and transmembrane migration of HCC cells. Through further analysis we noted that the expression levels of miR-124 in 11 HCC cell lines and other cancer cells was strikingly inversely correlated with the expression of integrin αV which is the driver molecule for the anchorage independence and migration of tumor cells^14^. This implied that miR-124 regulation of hepatocellular carcinoma cell migration might be related with the expression of integrin αV that is important in HCC cell migration and metastasis. We further investigated the possible regulation of integrin αV subunit expression by miR-124 and noted that miR-124 has two conserved binding sites on the 3′ untranslated region of transcription factor Sp1 mRNA. Through the luciferase reporter assay and binding site mutation, the results showed that miR-124 directly targeted Sp1 mRNA. MiR-124 may act together with miR-137 and miR-128 synergistically to regulate neural cells^25^. In this study we observed that miR-124 alone down-regulated the expression of Sp1 efficiently. Moreover, down-regulation of Sp1 by miR-124 subsequently inhibited integrin αV subunit expression since integrin αV subunit gene transcription relied on Sp1 as the major transcription factor in human hepatocellular carcinoma cells as demonstrated in our previous study^19^. Our current results further showed that overexpression of miR-124 reduced the expression levels of Sp1 and integrin αV subunit significantly. In functional studies, reintroduction of miR-124 dramatically repressed the migration and invasion of HCC *in vitro* and tumor metastasis *in vivo*. These results indicated that miR-124 functioned as a negative regulator or tumor suppressor for the cell growth and migration in HCC, which might be related to its repressing integrin αV subunit expression. Herein, we confirmed Sp1 as a direct target of miR-124, which played an important role in regulating integrin αV gene transcription.

These findings suggest that miR-124 plays an important role in the metastatic and/or invasive potential of HCC, which could be a potential therapeutic approach for HCC. Tumor metastasis is a combination of biological events which requires the flow of erroneous but precisely coordinated basic cellular activities such as cell migration-invasion, cell survival-apoptosis and cell proliferation. All of these processes require efficient regulation of cell attachment and detachment, which recruit integrin receptors in this flow of events^26^,^27^. In our previous studies, it had been demonstrated that the expression level of integrin αV was elevated in HCC specimens and hepatoma cells^17^,^19^. Through analysis using online algorithm TargetScan, we found that among 53 transcription factors screened Sp1 was not only predicted to match miR-124 target but also was the major transcription factor required for integrin αV gene promoter activation in HCC. Furthermore, the transcription expression of the integrin αV subunit gene is regulated by transcriptional factors Sp1^19^. In current study, we noted that the expression level of integrin αV significantly decreased in the cells overexpressing miR-124, but increased when the hepatoma cells were treated with miR-124 antagomir. Sp1, as a member of the Sp/KLF family, can be the target of miRNAs such as miR-223, miR-145, miR-129-5p and miR-429^17^,^28^,^29^,^30^. Meanwhile, post-translation modifications including phosphorylation, acetylation, glycosylation and proteolytic processing also significantly affect the activity of this protein, which can be an activator of integrin αV gene^31^. In our data, we identified Sp1 mRNA as the direct target of miR-124, and integrin αV gene as subsequent target in HCC. Overexpression of miR-124 significantly suppressed the luciferase reporter which contained Sp1 3′UTR and this suppression was interestingly abolished by the mutation of the miR-124 binding site in Sp1 3′UTR, and overexpression of miR-124 led to a significant reduction in Sp1 mRNA and protein levels, but the effect was reduced by miR-124 inhibitor.

Accumulating evidence has shed light on the critical role of miR-124 in the development and progression of cancers, including oncogenesis and proliferation^4^,^5^,^6^,^32^,^33^. These reports shows that the expression of miR-124 was reduced in carcinomas, which may result from multiple regulatory events, such as the methylation of CpG islands. Our data is consistent with these reports that miR-124 was down-regulated in HCC. The low level of miR-124 expression may be a candidate biomarker for further prospective molecular stratification of cancer patients possibly for prognosis prediction. Systemic delivery of miR-124 may perturb the hepatocyte growth and prevent hepatocellular carcinogenesis^34^. Our data further indicated that high level of miR-124 expression inhibited the wound healing, migration and invasion in HCC and suppressed integrin αV which is the malignant driver for anchorage independence. Ectopic overexpression of miR-124 significantly reduced the metastasis foci in either liver or lung tissues in nude mice experiments. High level of miR-124 expression in HCC directly resulted in low expression of Sp1, which subsequently suppressed integrin αV subunit gene expression. Therefore, our findings identified a novel pathway for miR-124 regulation of HCC metastasis, and miR-124 could possibly be an alternative strategy for controlling integrin αV expression in liver cancer and a viable anticancer therapeutic approach for HCC metastasis.

## Materials and Methods

### Cell culture and transfection

Human hepatocellular carcinoma cell lines SMMC-7721, BEL-7404 and non-tumor hepatocyte LO2 were obtained from Shanghai Institute of Biochemistry and Cell biology, Chinese Academy of Science. SMMC-7721 and BEL-7404 cells were infected with lentivirus containing miR-124 (SMMC-7721^miR-124^, BEL-7404^miR-124^) or control virus (SMMC-7721^control^, BEL-7404 ^control^). HCC cells LM3, Hep3B and HuH-7 were from Liver Cancer Institute of Zhongshan Hospital, Fudan University, Shanghai, China. Colorectal cancer cells SW480 and SW620 were from Shanghai Tumor Hospital (Fudan University, Shanghai, China). Hepatoma cells HepG2, SK-Hep-1, Lung cancer cells A549, cervical cancer HeLa and human embryonic kidney HEK-293T cells were obtained from ATCC. These cells were cultured at 37 °C with 5% CO_2_ in Dulbecco’s Modified Eagle’s Medium (DMEM, Gibco, CA, USA) supplemented with streptomycin (100 U/ML), penicillin (100 U/ML), and 10% newborn calf serum (PAA, Australia). The plasmid pLL 3.7-pre-miR-124 which carries green fluorescence protein (GFP) was constructed as described in our previous study^2^. Plasmids pLL-3.7-pre-miR-124 and psiCHECK-2 containing Sp1 3′UTR, mutated sequence or psiCHECK-2 control vector were co-transfected with Lipofectamine 2000 (Invitrogen, Carlsbad, CA, USA) at the ratio of 1:3 in weight. The miR-124 inhibitor and their scramble control oligonucleotides were synthesized and provided by Gene Pharma Co., Ltd (Shanghai, China). 1 OD inhibitor oligonucleotides were diluted in 125 μl water (20 μM) provided by the company.

### Wound healing assays

5.0 × 10^5^ cells were cultured into each well of 6-well plates after transfection, when cells grew to almost confluence, the cell monolayers were scratched by a sterile micropipette tip, and three wounds were made for each group. The scratched wound was photographed and measured every 24 hours under a microscope.

### Transmembrane migration assay

Human hepatocellular carcinoma SMMC-7721, Hep3B, HepG2, SK-Hep-1and non-tumor hepatocyte LO2 cells were transfected with miR-124 or control plasmids and 48 h after transfection, the cells were transferred into the upper chamber of the Millicell inserts with 8-μm pore size polyporous membrane (Millipore, Billerica, MA, USA) in a serum-free DMEM with a cell density of 5 × 10^6^/mL. As the chemoattractant, DMEM containing 10% fetal calf serum was added to the lower chamber. Cell migration was allowed to proceed for 12 hours at 37 °C in culture incubator. Invasion assay was performed in the same process but the insert membrane was pre-coated with 20 μg Matrigel and pre-incubated 1 hour to reconstitute a basement membrane. Tumor cell invasion was allowed to proceed for 24 hours at 37 °C in culture incubator. After incubation, non-migrated or uninvaded cells were removed from the inner part of the insert by using a cotton swab. Cells that had migrated or invaded through the membrane were fixed with methanol and stained with 0.5% crystal violet, imaged, and counted under a microscope.

### Western blotting analysis

SMMC-7721 or BEL-7404 cell, transfected with miR-124 construct or with miR-124 inhibitor oligonucleotide for 48 h, were harvested and lysed in lysis buffer (1%SDS containing 50 mM NaF, 1.5 mM Na_3_VO_4_, 0.5 mM PMSF). Proteins was fractionated on 10% SDS-polyacrylamide gels and transferred to a polyvinylidene fluoride membrane, followed by immunoblotting with the primary antibodies against Sp1 or integrin αV (Santa Cruz, CA, USA). Primary antibodies were then detected using horseradish peroxidase (HRP)-conjugated secondary antibodies and visualized using enhanced chemiluminescence detection kit (Pierce, Thermo, Rockford, IL, USA). The images were captured by an E/M CCD camera (Tenon, Shanghai, China), and the density of protein bands was analyzed and summarized.

### RNA extraction and polymerase chain reaction (PCR)

Total RNA was isolated from SMMC-7721 or BEL-7404 cells using TRIzol reagent (Life Technologies, Garlsbad, CA, USA), and the RNA extract was subjected to reverse transcription (RT) reaction using the M-MLV reverse transcriptase (TAKARA, Dalian, China) according to the manufacturer’s instructions. The polymerase chain reaction was performed in a thermocycler (FXC-connect, Bio-Rad, Singapore). The relative expression levels of the target genes were evaluated on the base of glyceraldehyde-3-phosphate dehydrogenase (GAPDH), a reference gene, with the principle of SYBR Green technology. The data analyses were performed using the 2^−ΔΔCt^ method.

### Luciferase Reporter Assay

HeLa or HEK-293T Cells were co-transfected with the indicated plasmid and a dual luciferase reporter constructs containing Sp1 mRNA 3′ untranslated region cloned into the 3 prime of renilla luciferase gene of psiCHECK-2 vector. Fire fly and renilla luciferase activities were determined using the Dual-Luciferase Reporter Assay System (Promega, Madison, WI, USA) with an illuminometer (Lumat LB 9507, Berthold, Germany) according to manufacturer’s instructions. Data were calculated by normalizing luminescence of Fire fly luciferase to that of Renilla luciferase.

### *In vivo* metastasis assays

After lentivirus infection and selection, SMMC-7721 cells that stably expressed miR-124 (SMMC-7721^MiR-124^) and control cells (SMMC-7721^Mock^) (5 × 10^6^) were injected into 6-week-old female nude mice through the tail veins (6 mice/group) for the assessment of *in vivo* metastasis. Four weeks after injection, the livers and lungs were isolated and fixed in 10% buffered formalin for hematoxylin and eosin (H&E) staining. The numbers of the intrahepatic metastatic foci (visible and micro) in the liver and lungs were then counted. All experiments involving animals were performed according to the Animals (Control of Experiments) Ordinance and approved by the Animal Ethics Committee of Fudan University Shanghai Medical College with the permit number of 20140226-001. The BALB/C athymic nude mice for this experiments were from SLAC LAB ANIMAL LTD, CO. (Shanghai, China). All the animals used in this study were approved by Shanghai Municipal Government with the permit number of 2015000510941.

### Bioinformatics analysis

The targets of miR-124 were analyzed using four online algorithms including miRanda, TargetScan, PicTar and microRNA.org. All the transcription factors screened were the predicted targets of miR-124 and all of them were important in HCC. The network for targeted transcription factors and integrin αV gene was made by Cytoscape (version 3.3.0, NRNB, US, http://www.cytoscape.org).

### HCC sample analysis

The human hepatocellular carcinoma data were downloaded and analyzed from TCGA (The Cancer Genome Atlas, https://genome-cancer.ucsc.edu/proj/site/hgHeatmap/). The data included both gene expression (IlluminaHiSeq) and miRNA expression (IlluminaHiseq). In 58 cases both integrin αV and miR-124 expression were measured in the same sample.

### Statistical analysis

Data are presented as means ± SD. Statistical differences were measured using one-way ANOVA and Student’s t test. Chi-square test was used for the analysis of ratio difference.

## Additional Information

**How to cite this article**: Cai, Q. Q. *et al*. MiR-124 inhibits the migration and invasion of human hepatocellular carcinoma cells by suppressing integrin αV expression. *Sci. Rep.* **7**, 40733; doi: 10.1038/srep40733 (2017).

**Publisher's note:** Springer Nature remains neutral with regard to jurisdictional claims in published maps and institutional affiliations.
